# External validation and update of a prognostic model to predict mortality in hospitalized adults with RSV: A retrospective Dutch cohort study

**DOI:** 10.1002/jmv.25568

**Published:** 2019-08-28

**Authors:** Laura M. Vos, Jan Jelrik Oosterheert, Andy I. M. Hoepelman, Louis J. Bont, Frank E. J. Coenjaerts, Christiana A. Naaktgeboren

**Affiliations:** ^1^ Department of Infectious Diseases, University Medical Centre Utrecht Utrecht University Utrecht The Netherlands; ^2^ Department of Pediatric Infectious Diseases, Wilhelmina Children's Hospital, University Medical Centre Utrecht Utrecht University Utrecht The Netherlands; ^3^ Department of Medical Microbiology, University Medical Centre Utrecht Utrecht University Utrecht The Netherlands; ^4^ Julius Centre for Health Sciences and Primary Care, University Medical Centre Utrecht Utrecht University Utrecht The Netherlands

**Keywords:** adults, external validation, mortality, prognostic, respiratory infection, RSV

## Abstract

Respiratory syncytial virus (RSV) causes significant mortality in hospitalized adults. Prediction of poor outcomes improves targeted management and clinical outcomes. We externally validated and updated existing models to predict poor outcome in hospitalized RSV‐infected adults. In this single center, retrospective, observational cohort study, we included hospitalized adults with respiratory tract infections (RTIs) and a positive polymerase chain reaction for RSV (A/B) on respiratory tract samples (2005‐2018). We validated existing prediction models and updated the best discriminating model by revision, recalibration, and incremental value testing. We included 192 RSV‐infected patients (median age 60.7 years, 57% male, 65% immunocompromised, and 43% with lower RTI). Sixteen patients (8%) died within 30 days. During hospitalization, 16 (8%) died, 30 (16%) were admitted to intensive care unit, 21 (11%) needed invasive mechanical ventilation, and 5 (3%) noninvasive positive pressure ventilation. Existing models performed moderately at external validation, with *C*‐statistics 0.6 to 0.7 and moderate calibration. Updating to a model including lower RTI, chronic pulmonary disease, temperature, confusion and urea, increased the *C*‐statistic to 0.76 (95% confidence interval, 0.61‐0.91) to predict in‐hospital mortality. In conclusion, existing models to predict poor prognosis among hospitalized RSV‐infected adults perform moderately at external validation. A prognostic model may help to identify and treat RSV‐infected adults at high‐risk of death.

## Highlight

- Prediction of poor outcomes among RSV‐infected adults may improve targeted management and clinical outcomes.
- Existing models to predict poor prognosis among hospitalized RSV‐infected adults perform moderately at external validation.
- A model including lower RTI, chronic pulmonary disease, temperature, confusion and urea, had a *C*‐statistic of 0.76 (95%CI 0.61‐0.91) to predict in‐hospital mortality.

## 1 INTRODUCTION

There is increasing evidence that respiratory syncytial virus (RSV) is a common cause of respiratory tract infections (RTI) in adult patients,^1^ often with a complicated course of disease.^2^, ^3^, ^4^, ^5^ Among hospitalized elderly ≥65 years of age mortality is as high as 8%,^2^ but among high‐risk groups as patients with chronic heart or lung disease, long‐term care facility residents and immunocompromised patients as lung or hematopoietic cell transplant (HCT) recipients, RSV may even lead to mortality rates over 50%.^2^, ^3^, ^6^, ^7^ With the widespread implementation of rapid tests for respiratory viruses in‐hospital care settings, early detection of RSV enables early treatment with either aerosolized or oral ribavirin^6^, ^8^, ^9^, ^10^, ^11^, ^12^, ^13^, ^14^ and future medicaments as fusion protein inhibitors (eg, presatovir), nucleoside inhibitors (eg, lumicitabine),^15^ and viral replication lowering immunoglobulins (eg, palivizumab), which might have an additional positive effect to ribavirin.^11^, ^16^, ^17^, ^18^ Ideally, in light of effectivity and potential side effects, treatment should be targeted to patients at the highest risk of a life‐threatening infection. Identification of RSV‐infected patients at high‐risk of death is therefore necessary to improve targeted therapy and clinical outcomes. In addition, the prediction of individual prognosis improves decision making on the necessity to apply supportive in‐hospital management as intensive care unit (ICU) admission and strict isolation procedures.^3^ However, a validated prognostic model to identify adult patients with a high mortality risk is not available. Therefore, we aimed to establish factors associated with poor prognosis and externally validate and update existing models to predict mortality in hospitalized RSV‐infected adults.

## 2 METHODS

### 2.1 Study population

We performed a single center cohort study to validate prognostic models for poor outcomes in hospitalized adults with RSV. In the validation cohort we included adult patients (≥18 years) with a laboratory confirmed community acquired RSV‐infection between January 2005 and April 2018 who were admitted to the University Medical Center Utrecht (UMCU), a 1042‐bedded tertiary care hospital in the central region of The Netherlands. We excluded patients with hospital acquired RSV‐infection (RSV result >48 hours after admission). When patients had more than one hospitalized RSV‐infection episode during the study period, only the first episode was included. RSV positive patients were identified retrospectively using the microbiology laboratory database of the UMCU. During the inclusion period, in‐house reverse transcription polymerase chain reaction (RT‐PCR) was used for detection of RSV and other respiratory viral pathogens^19^, ^20^ in respiratory tract specimens. A positive RSV result was defined as having a cycle time (Ct) value les than 40.^21^ For immunocompromised patients, the conventional in‐house RT‐PCR was replaced by a qualitative RT‐PCR‐the FilmArray respiratory viral panel version 1.7 (BioFire Diagnostics, Salt Lake City)^22^ from November 2016 onwards. Collection of predictor and outcome variables was performed retrospectively from the electronic patient files. This study was assessed by the medical ethics committee of the UMCU (METC protocol no 18‐410/C). Due to the retrospective nature of the study, informed consent was not required. Results were reported to conform with the transparent reporting of a multivariable prediction model for individual prognosis or Diagnosis (TRIPOD) statement (Table S1).^23^

### 2.2 External validation

We searched available literature on predictive models for RSV prognosis in the MEDLINE. We aimed to validate models predicting mortality, but also included studies using a composite outcome including mortality. For the external validation, we applied the included original prognostic models to our study cohort exactly as they were published, with similar definitions of predictor variables and outcomes (Table S2).^24^, ^25^, ^26^, ^27^ If the intercept from the original model was not reported, we calculated a new intercept by recalibration. We compared the discriminative ability of the models using the Harrell's *C*‐statistic. Calibration of the models was assessed in calibration plots.^28^, ^29^, ^30^

### 2.3 Model update

We selected the model with the best discrimination and calibration for further updating.^24^ In view of increasingly shorter turnaround times of molecular diagnostics and increased effectiveness of antiviral treatment when given at an early stage,^6^ we first removed any eventual predictors that could not be assessed at the time of presentation/RSV diagnosis, eg, bacterial coinfection. Furthermore, we replaced binary predictors with continuous to avoid loss of information, eg, temperature instead of fever. Next, we recalibrated the calibration slope and intercept by refitting this adapted model in the validation cohort. Consequently, we tested the incremental value of the model by adding objectively assessable predefined predictor variables (age, gender, urea, confusion, cardiovascular comorbidities, immunocompromised status, and the number of other comorbidities), based on the existing prognostic models for poor outcomes in patients with positive influenza virus.^26^, ^27^, ^31^ We performed backward variable selection based on the Akaike information criterion and Occam's razor principle. Finally, we performed internal validation with optimism correction by bootstrap.^32^ Discrimination and calibration of this final updated and extended model was assessed for in‐hospital mortality, 30‐day mortality and a composite outcome consisting of in‐hospital death, ICU‐admission and/or need for mechanical ventilation separately. Furthermore, we performed a decision curve analysis to provide insight into the range of predicted risks for which the final model results in better clinical decision making, eg, is better than either classifying all or none of the patients as having the outcome.^33^

### 2.4 Statistical analysis

For the validation cohort, we accounted for missing values of predictors using a multiple imputation model including baseline characteristics, predictors, and outcome variables. Results shown are pooled from the 10 multiple imputed datasets.^34^ Calibration plots were derived from all 10 multiple imputed datasets combined. Analyses were performed by the SPSS version 25 (IBM Corp) and the rms, mice, survival, and rmda packages of R‐3.1 for Windows (http://cran.r-project.org).

## 3 RESULTS

### 3.1 Validation cohort

**Figure 1:**
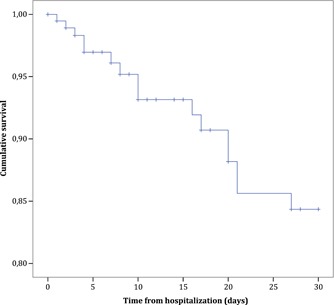
Kaplan‐Meier survival curve of 192 adults hospitalized with RSV‐infection. RSV, respiratory syncytial virus

We included 192 hospitalized, RSV‐infected adult patients. Demographics and characteristics of the included patients are displayed in Table 1. The median age was 60.7 (interquartile range [IQR], 50.8‐69.2) years. In total, 125 patients (65.1%) were immunocompromised, of whom 42 patients were HCT and 34 solid organ transplant recipients. At presentation, 83 patients (43.2%) were diagnosed with a lower RTI. After hospitalization, 16 patients (8.3%) died during their hospital stay. In‐hospital mortality was not different between immunocompromised (n = 9) and immunocompetent (n = 7) patients (odds ratio [OR], 0.62 [95% confidence intervals [CI], 0.22‐1.73]) or between HCT (n = 2) and solid organ transplant (n = 3) recipients (OR, 0.52 [95%CI, 0.08‐3.29]). At 30 days, 16 patients had died (Figure 1). During hospitalization, 30 patients (15.6%) were admitted to the ICU, of whom 21 patients needed invasive mechanical ventilation, five needed noninvasive positive pressure ventilation and four needed no ventilator support. Of all ICU‐admitted patients, 23 were admitted to the ICU within the first 48 hours of admission. The median length of hospital stay was 5 days (IQR, 3‐10) and 77 patients (40.1%) had a hospital stay ≥7 days. In total, 147 patients (76.6%) were treated with antibiotics empirically and 25 patients (13.0%) were treated with oral ribavirin, of whom 18 for ≥7 days. Over the years, the annual number of included patients increased, with no clear changes in in‐hospital mortality rate (Figure S1).

**Table 1:** Demographics and characteristics of included patients in validation cohort (n = 192)

Characteristics	Validation cohort (n = 192), n (%) or median (IQR)
Demographics	
Age, y	60.7 (50.8‐69.2)
Male gender	110 (57.3%)
Immunocompromised^a^	125 (65.1%)
Smoking^a^	100 (52.1%)
Chronic pulmonary disease^a^	67 (34.9%)
Disease characteristics at presentation	
Symptom duration before presentation, d	3.4 (2.0‐7.0)
Confusion^a^	17 (8.9%)
Heart rate, beats per minute	100 (88‐115)
Ear‐based temperature, °C	37.8 (37.1‐38.9)
Systolic blood pressure, mmHg	130 (115‐145)
Diastolic blood pressure, mmHg	75 (65‐85)
Breathing frequency, breaths per minute	20 (16‐26)
Saturation, %^b^	95 (92‐97)
Meeting sepsis criteria, qSOFA score ≥2^c^	15 (7.8%)
Laboratory findings at presentation	
pO_2_ arterial blood gas, mmHg^b^	71 (58‐94)
pH arterial blood gas	7.46 (7.39‐7.50)
Hemoglobin, mmol/L	7.9 (6.8‐8.6)
Thrombocytes (×10^9^/L)	203 (128‐257)
Leukocytes (×10^9^/L)	8.3 (4.7‐12.1)
Lymphocytes (×10^9^/L)	1.3 (0.6‐2.5)
Neutrophils (×10^9^/L)	5.5 (2.5‐9.5)
C‐reactive protein (mg/L)	60 (21‐136)
Sodium, mmol/L	135 (133‐138)
Urea, mmol/L	7.1 (4.8‐10.7)
Results from other diagnostics at presentation	
Ct value RSV, quantitative RT‐PCR	29.1 (25.2‐33.8)
Lower RTI^a^	83 (43.2%)
Bacterial coinfection^a^	81 (42.2%)

Abbreviations: Ct, cycle time; IQR, interquartile range; pO_2_, partial pressure of oxygen; RSV, respiratory syncytial virus; RTI, respiratory tract infection; RT‐PCR, reverse transcription polymerase chain reaction.

^a^ Table S2 for definitions.

^b^ Not always clear if taken with or without oxygen replacement therapy.

^c^ qSOFA criteria: altered mental status (Glasgow Coma Scale <15), respiratory rate ≥22, systolic blood pressure ≤100.

### 3.2 External validation

**Figure 2:**
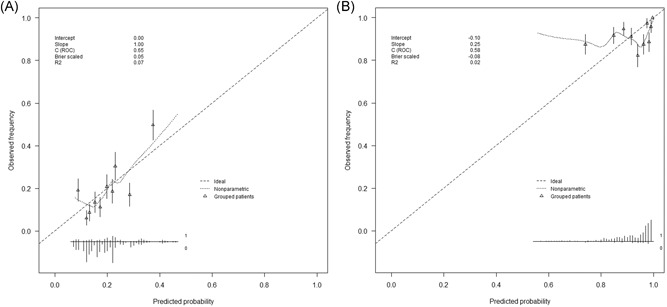
Calibration plots of original prognostic models. A, Predicted probabilities determined by the original model of Park et al^24^ (chronic pulmonary disease, lower RTI, temperature ≥38°C, bacterial coinfection)—with a recalibrated intercept—plotted against the observed frequency of the primary outcome (ICU‐admission, need for mechanical ventilation, and/or in‐hospital death) divided in 10‐deciles of predicted probabilities. B, Predicted probability of 30‐day survival determined by the original model by Lee et al^25^ (age >75, male gender, pneumonia, need for ventilatory support, bacterial coinfection, and urea) plotted against the actually observed 30‐day survival. ICU, intensive care unit; RTI, respiratory tract infection

We found five studies that developed a prognostic model for hospitalized RSV‐infected adult patients, of which two to predict mortality^24^, ^25^ and three to predict disease progression to a lower RTI^34^, ^35^, ^36^, ^37^ (Figure S2). The two models to predict mortality were included for external validation. A detailed overview of these two models is shown in Table 2. The first study, of Park et al^24^, developed a logistic regression model to predict in‐hospital death, ICU‐admission and/or the need for mechanical ventilation. In our validation cohort, 36 patients (18.8%) met this composite outcome (vs 15.0% in the original study; *P* = .300). When applying the original logistic regression model of Park et al^24^ (with a recalibrated intercept) to our validation cohort, the C‐statistic was 0.65 (95% CI, 0.55‐0.76) for this composite outcome. The model showed good calibration when plotting predicted against observed poor outcomes (Figure 2A). The second study, of Lee et al^25^, developed a survival model to predict 30‐day mortality. In our validation cohort, 16 patients (8.3%) died within 30 days (vs 9.1% in the original study; *P* = .735). When applying the original Cox proportional hazards model of Lee et al^25^ with 30‐day mortality as outcome, the *C*‐statistic was 0.61 (95% CI, 0.49‐0.73). The calibration plot of this model plotting predicted against observed survival at 30 days, showed reasonable calibration (Figure 2B).

**Table 2:** Characteristics of included models

Study characteristics	Park et al^24^	Lee et al^25^
Study population	Hospitalized adults with an RSV RTI presenting at the emergency department (n = 227); 133 (59%) community acquired, 94 (41%) healthcare‐associated. In total, 84 (37%) patients were immunocompromised (25 solid organ recipients, 9 patients, with HCT 50 using immunosuppressants/ corticosteroids) and 42 (19%) had a chronic pulmonary disease	Hospitalized adults with an RSV RTI (n = 607). In total, 83 (13.7%) patients were immunocompromised and 216 (36%) had a chronic pulmonary disease
	Exclusion: ≤18 y, outpatient treatment, RSV diagnosis >48 h after admission, concurrent infections at other sites	Exclusion: none
Primary outcome	Life‐threatening RSV‐infection (admission to ICU, need for ventilator care or in‐hospital death; n = 34, 15.0%)	30‐d mortality (n = 55, 9.1%), 60‐d mortality (n = 72, 11.9%)
Patient identification and data collection	Identification using RSV positive PCR assays; retrospective data collection	Identification of RSV positive viral antigen immunofluorescence assay tests; retrospective data collection
Inclusion location	ED of a 2700‐bed tertiary care hospital in Seoul, South Korea	Three acute care, general public hospitals in Hong Kong, China
Inclusion period	October 2013‐September 2015	January 2009‐December 2011
Modeling technique	Multivariable logistic regression analysis with stepwise backward variable selection	Multivariable Cox proportional hazards analysis with stepwise backward variable selection
Variable selection for multivariable analysis	Variables with *P* ≤ .05 in univariate analyses of association with life‐threatening infection. Exclusion of variables in causal pathway (confusion, saturation); subjective symptoms (dyspnea); correlated variables (smoking history, correlated with chronic pulmonary disease)	Variables with *P* ≤ .1 in univariate analyses of association with mortality. Inclusion of demographics, comorbidities, cardiorespiratory complications, ventilation requirement, bacterial superinfection, and corticosteroid use
Variables included in multivariable analysis^a^	Lower RTI, chronic pulmonary disease, bacterial coinfection, fever ≥38°C, rhinorrhoea, CRP, procalcitonin, RSV type A and B, antimicrobial use^b^, ribavirin use^b^	Age, gender, major systemic comorbidity, chronic pulmonary disease exacerbation, cardiovascular complications, pneumonia, need for ventilatory support, bacterial coinfection, urea, total white cell count, systemic corticosteroid use
Variables in final model^a^	Lower RTI, chronic pulmonary disease, bacterial coinfection, fever ≥38⁰C	Age >75 y, male gender, pneumonia, need for ventilatory support, bacterial coinfection, urea
Missing data handling	Not described	Not described

Abbreviations: CRP, C‐reactive protein; DFA, direct fluorescent antibody; ED, emergency department; HCT, hematopoietic cell transplant; ICU, intensive care unit; PCR, polymerase chain reaction; RSV, respiratory syncytial virus; RTI, respiratory tract infection.

^a^ Table S2 for definitions.

^b^ No further definition or details given.

### 3.3 Model update

**Figure 3:**
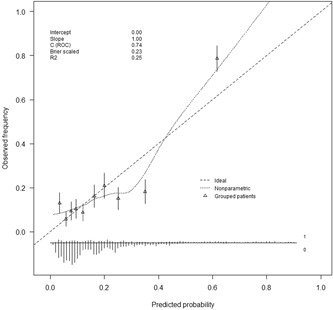
Calibration plot of updated and extended prognostic model of Park et al^24^ (with predictors chronic pulmonary disease, lower RTI, temperature, confusion and urea) for the prediction of ICU‐admission, need for mechanical ventilation and/or in‐hospital death. ICU, intensive care unit; RTI, respiratory tract infections

**Figure 4:**
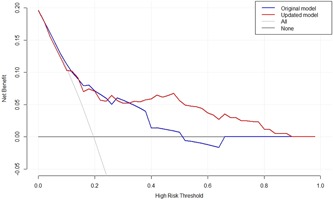
Decision curve analysis showing the net benefit curve of the original model of Park et al^24^ (in blue) and of the final updated prognostic model (in red) for the composite poor outcome (ICU‐admission, need for mechanical ventilation and/or in‐hospital death). The horizontal gray line is the net benefit when all RSV‐infected hospitalized adults are considered as not having the poor outcome; vertical gray line is the net benefit when all RSV‐infected hospitalized adults are considered as having the poor outcome. The higher the net benefit (blue line) at any given threshold, the better the model performs. Example: with a risk threshold of 25% (threshold above which we would treat), the net benefit (derived from the true positives and true negatives) is 5.33 per 100 patients when using the original model of Park et al^24^ and 5.95 when using the updated model. RSV, respiratory syncytial virus; ICU, intensive care unit

We updated and extended the model of Park et al,^24^ which was the best performing model in terms of discrimination and calibration, by performing variable revision, recalibration of the regression coefficients and incremental value testing. The final model included three predictors from the original model of Park et al^24^, eg, lower RTI, chronic pulmonary disease and temperature, and two newly added predictors, eg, urea and confusion. The final updated, optimism corrected model had a *C*‐statistic of 0.76 (95% CI, 0.61‐0.91) for the prediction of in‐hospital mortality, a *C*‐statistic of 0.73 (95% CI, 0.59‐0.88) for prediction of 30‐day mortality and a C‐statistic of 0.74 (95% CI, 0.64‐0.84) for prediction of in‐hospital mortality and/or ICU‐admission and/or need for mechanical ventilation. The updated model showed good calibration for the composite outcome (Figure 3). Results of the decision curve analysis of the updated model is shown in Figure 4. For the whole range of predicted risks, the updated prognostic model showed a positive net benefit. However, only with a risk threshold—eg, a predicted risk threshold that can be used for decision‐making regarding therapy—above 40%, the updated model improved the net benefit as compared to the original model of Park et al.^24^

## 4 DISCUSSION

We showed that hospitalized, RSV‐infected adults had an 8% in‐hospital and 8% 30‐day mortality rate. We validated and updated models to predict poor outcome in these patients at the time of RSV diagnosis. This model can be used to develop a risk score or decision tool to guide decisions on treatment with ribavirin, immune globulins, and other antivirals and on site‐of‐care and strict isolation procedure decisions, as is already common practice for influenza virus.^38^ These interventions might improve clinical outcomes for patients with life‐threatening disease.

To our knowledge, this is one of the largest studies in RSV‐infected adult patients in a hospital care setting. We found a high percentage of 8% in‐hospital mortality, which is in line with 8% to 9% mortality rates reported in former publications.^2^, ^6^, ^24^, ^25^ This high mortality rate underlines the great importance of targeted treatment for these patients. Also, this is the first study to externally validate existing models to predict poor prognosis in RSV‐infected hospitalized adult patients, and allows for a head‐to‐head comparison of two published models. Unfortunately, model performance in the development cohorts was not described,^30^ but the poor to moderate discriminative abilities of both models in our validation cohort with *C*‐statistics under 0.7 with CI close to or overlapping 0.5, indicate that both models are not suitable for use in daily practice, at least not in our Dutch tertiary care setting. To some extent, the poor predictions in our validation cohort might be caused by differences in average values of various predictors and administered treatments as compared to the development studies.^30^ Geographical validation is also very likely to have played a role and affected the performance of these models in our validation cohort,^30^ since both development studies were performed in Asia. Temporal and domain validation—with 37%^24^ and 14%^25^ vs 65% immunocompromised patients for example‐might also have resulted in lower prediction accuracy of the two models, although the proportions of our patients who met the outcomes were quite similar to the development studies.^30^ Another, maybe the most important factor that might have caused the moderate performance of both models at external validation, was the relatively small cohort in which these models were developed, with a rather low number of events causing overfitted estimations of predictor effects.^32^ If internal validation methods as bootstrap would have been performed after development of these models, poor external validation might have been foreseen.^32^, ^39^

During the model update, the viral load (eg, Ct value) of RSV was not considered a useful predictor. First, the interpretation of single viral load measurements is difficult. Not only are viral loads of respiratory viruses highly dependent on variation in sampling timing, location and technique, they also rise and drop rapidly and it is known that symptoms mostly follow the highest peak in viral load.^40^, ^41^ Second, since more and more rapid qualitative molecular methods are implemented, viral loads will not always be available.

The updated model of Park showed good discrimination and calibration and the net benefit of this updated model was positive for the whole range of predicted risks. For clinical practice, to be able to use this prediction model as decision tool for RSV treatment, a new external validation and a well‐considered harm‐benefit based treatment threshold are needed. The more convincing the benefits of RSV treatment on improved clinical patient outcomes and hospital management, and the lower the potential harms—serious side effects, complications, and increased costs, the lower the appropriate treatment threshold. When a consensus based threshold is determined, the positive predictive value of the model determines the positive effect of implementing such a model in clinical practice, eg, the benefit of implementing the model over treating all or none of the patients.

In addition to the fact that we had a large cohort and performed external validation according to current guidelines, we performed a model update according to the TRIPOD statement,^23^ including internal validation procedures.^30^ However, some limitations of our study need to be addressed. First, we had a limited amount of patients with the primary outcome. For studies validating prognostic models, there is no solid sample size recommendation, but it is recommended to consider at least the number of predictors, the total sample size and the event fraction.^42^, ^43^, ^44^ The low number of events in our study might have resulted in biased and less precise performance measures, which is also indicated by the broad CI of the reported *C*‐statistics. Second, the performance of routine clinical care diagnostic RSV tests was non‐standardized and subjected to change during the 14‐year study period, bearing the risk of selective patient inclusion with more severely ill patients and the risk of missed RSV diagnoses. Third, over the years, increased awareness for the disease burden of RSV in adult patients might have led to and more targeted treatment with a positive effect on the prognosis of RSV‐infected patients. Increased awareness might also have resulted in more frequent testing for RSV. Unfortunately, due to the absence of the number of adults tested for RSV, we cannot confirm this hypothesis based on our data. Finally, we included relatively many immunocompromised patients, making results potentially less generalizable to other settings as nonacademic hospitals.

In conclusion, hospitalized RSV‐infected adults have a very poor prognosis with 8% in‐hospital and 8% 30‐day mortality. This poor prognosis could be improved by targeting RSV treatment with ribavirin, immune globulins, future antiviral treatment options, site‐of‐care decisions, and strict isolation procedures for patients at highest risk of serious complications. Existing models to predict mortality in these patients perform moderately or poor at external validation. An updated model including chronic pulmonary disease, lower RTI, confusion, temperature, and urea, however, reasonably predicts which RSV‐infected patients are at highest risk of poor prognosis. Implementation of this prediction model in clinical practice could improve clinical outcomes of high‐risk patients, without putting low‐risk patients at an unnecessary treatment risk.

## CONFLICT OF INTERESTS

The authors declare that there are no conflict of interests.

## AUTHOR CONTRIBUTIONS

LV, CN, and JO designed the study. LV collected the clinical data of included patients and performed all formal analyses with close involvement of CA. All authors contributed to reviewing and editing of the manuscript. Dr. Bont has regular interaction with pharmaceutical and other industrial partners (AbbVie, MedImmune, Janssen, the Bill and Melinda Gates Foundation, MeMed Diagnostics, Regeneron, Ablynx, Bavaria Nordic, MabXience and Novavax). He has not received personal fees or other personal benefits.

## Supporting information

Supplementary information

Supplementary information

Supplementary information
