# Supplementary figures and images for: External validation and update of a prognostic model to predict mortality in hospitalized adults with RSV: A retrospective Dutch cohort study
Source: J Med Virol. 2019 Aug 28;91(12):2117–24. doi: 10.1002/jmv.25568 (PMC6851775; doi:10.1002/jmv.25568)

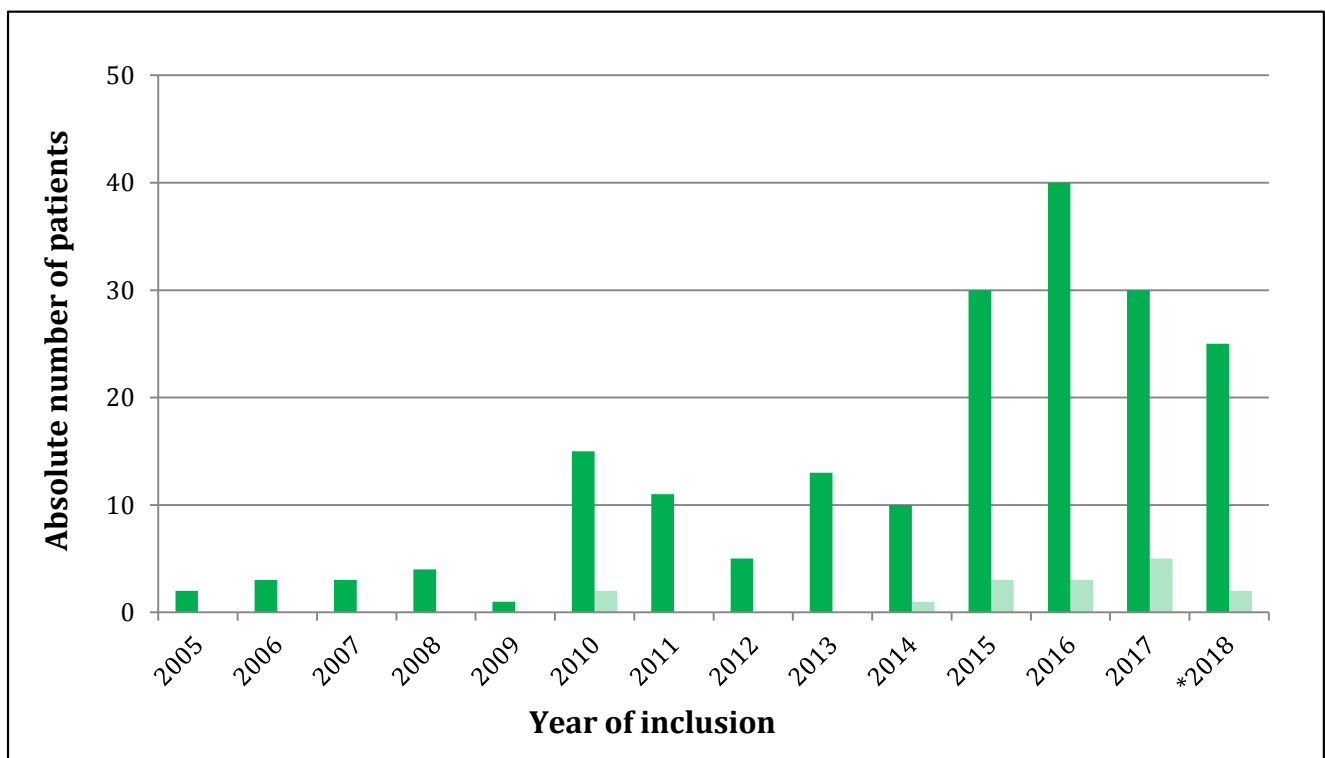

Supplement: Supplementary file 1 — Supplementary information [file JMV-91-2117-s001.pdf]

**Search [prognostic] models for [RSV] in [adults]<sup>a</sup>**

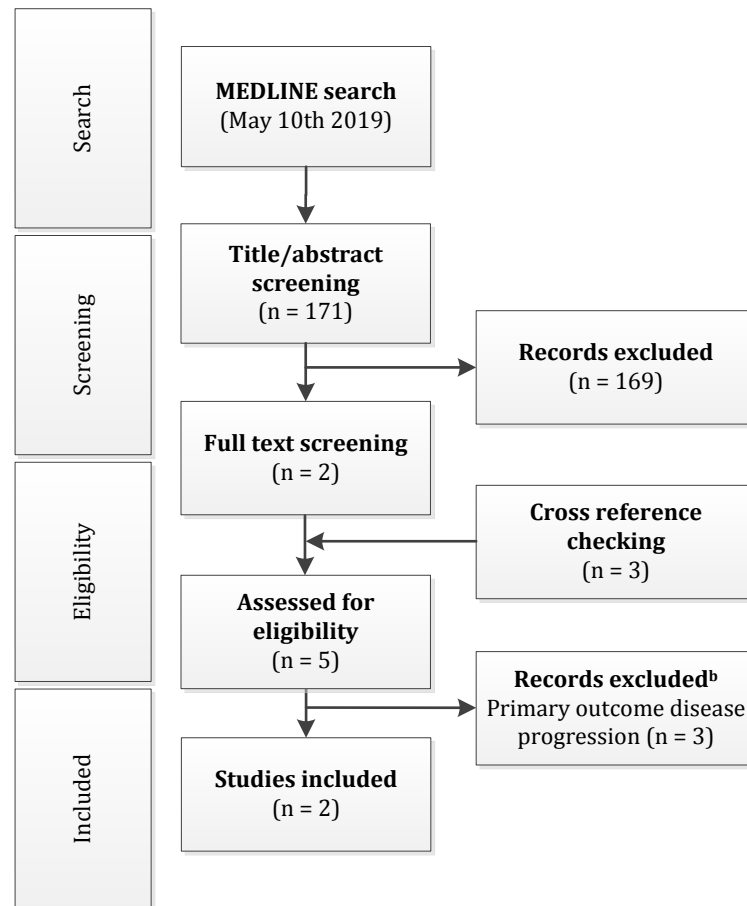

Supplement: Supplementary file 2 — Supplementary information [file JMV-91-2117-s002.pdf]
