# Supplementary material for: External validation and update of a prognostic model to predict mortality in hospitalized adults with RSV: A retrospective Dutch cohort study
Source: J Med Virol. 2019 Aug 28;91(12):2117–24. doi: 10.1002/jmv.25568 (PMC6851775; doi:10.1002/jmv.25568)
Supplement: Supplementary file 3 — Supplementary information [file JMV-91-2117-s003.docx]

**Supplementary Table 1.** *Checklist of Items to Include When Reporting a Study Developing or Validating a Multivariable Prediction Model for Diagnosis or Prognosis.*

| **Section/ topic** | **Item** | **Development (D) or validation (V)** | **Checklist item** |
| --- | --- | --- | --- |
| Title | 1 | D;V | Identify the study as developing and/or validating a multivariable prediction model, the target population, and the outcome to be predicted. |
| Abstract | 2 | D;V | Provide a summary of objectives, study design, setting, participants, sample size, predictors, outcome, statistical analysis, results, and conclusions. |
| Background and objectives | 3a | D;V | Explain the medical context (including whether diagnostic or prognostic) and rationale for developing or validating the multivariable prediction model, including references to existing models. |
|  | 3b | D;V | Specify the objectives, including whether the study describes the development or validation of the model, or both. |
| Source of data | 4a | D;V | Describe the study design or source of data (e.g., randomized trial, cohort, or registry data), separately for the development and validation datasets, if applicable. |
|  | 4b | D;V | Specify the key study dates, including start of accrual; end of accrual; and, if applicable, end of follow-up. |
| Participants | 5a | D;V | Specify key elements of the study setting (e.g., primary care, secondary care, general population) including number and location of centres. |
|  | 5b | D;V | Describe eligibility criteria for participants. |
|  | 5c | D;V | Give details of treatments received, if relevant. |
| Outcome | 6a | D;V | Clearly define the outcome that is predicted by the prediction model, including how and when assessed. |
|  | 6b | D;V | Report any actions to blind assessment of the outcome to be predicted. |
| Predictors | 7a | D;V | Clearly define all predictors used in developing the multivariable prediction model, including how and when they were measured. |
|  | 7b | D;V | Report any actions to blind assessment of predictors for the outcome and other predictors. |
| Sample size | 8 | D;V | Explain how the study size was arrived at. |
| Missing data | 9 | D;V | Describe how missing data were handled (e.g., complete-case analysis, single imputation, multiple imputation) with details of any imputation method. Describe how predictors were handled in the analyses. |
| Statistical analysis methods | 10a | D | Describe how predictors were handled in the analyses. |
|  | 10b | D | Specify type of model, all model-building procedures (including any predictor selection), and method for internal validation. |
|  | 10c | V | For validation, describe how the predictions were calculated. |
|  | 10d | D;V | Specify all measures used to assess model performance and, if relevant, to compare multiple models. |
|  | 10e | V | Describe any model updating (e.g., recalibration) arising from the validation, if done. |
| Risk groups | 11 | D;V | Provide details on how risk groups were created, if done. |
| Development vs validation | 12 | V | For validation, identify any differences from the development data in setting, eligibility criteria, outcome, and predictors. |
| Participants | 13a | D;V | Describe the flow of participants through the study, including the number of participants with and without the outcome and, if applicable, a summary of the follow-up time. A diagram may be helpful. |
|  | 13b | D;V | Describe the characteristics of the participants (basic demographics, clinical features, available predictors), including the number of participants with missing data for predictors and outcome. |
|  | 13c | V | For validation, show a comparison with the development data of the distribution of important variables (demographics, predictors, and outcome). |
| Model development | 14a | D | Specify the number of participants and outcome events in each analysis. |
|  | 14b | D | If done, report the unadjusted association between each candidate predictor and outcome. |
| Model specification | 15a | D | Present the full prediction model to allow predictions for individuals (i.e., all regression coefficients, and model intercept or baseline survival at a given time point). |
|  | 15b | D | Explain how to use the prediction model. |
| Model performance | 16 | D;V | Report performance measures (with CIs) for the prediction model. |
| Model updating | 17 | V | If done, report the results from any model updating (i.e., model specification, model performance). |
| Limitations | 18 | D;V | Discuss any limitations of the study (such as non-representative sample, few events per predictor, missing data). |
| Interpretation | 19a | V | For validation, discuss the results with reference to performance in the development data, and any other validation data. |
|  | 19b | D;V | Give an overall interpretation of the results, considering objectives, limitations, results from similar studies, and other relevant evidence. |
| Implications | 20 | D;V | Discuss the potential clinical use of the model and implications for future research. |
| Supplementary information | 21 | D;V | Provide information about the availability of supplementary resources, such as study protocol, Web calculator, and datasets. |
| Funding | 22 | D;V | Give the source of funding and the role of the funders for the present study. |

**Supplementary Table 2.** *Definitions predictor variables.*

| **Predictor variable** | **Definition** |
| --- | --- |
| Immunocompromised status | Use of corticosteroids (prednisone or equivalent with a cumulative dose of >700mg); use of anti-CD20 therapy, biologicals, methotrexate, azathioprine and/or mercaptopurine within the last 6 months; having received an autologous/allogenic stem-cell transplantation; and neutropenia (<0.5x109/L), (functional) hypo/asplenia, CD4-penia (<200 cells/mm3), hypogammaglobinaemia and/or another primary immunodeficiency at the time of presentation. |
| Systemic corticosteroid use | Use of intravenous hydrocortisone or oral prednisolone. |
| Chronic pulmonary disease | Chronic obstructive pulmonary disease (COPD); bronchiectasis; cystic fibrosis (CF); interstitial lung fibrosis; pneumoconiosis and/or bronchopulmonary dysplasia. |
| Cardiovascular diseases | Myocardial infarction; angina pectoris; heart failure; cerebrovascular accident (CVA); transient ischemic attack (TIA); aortal aneurysm; pulmonary embolism; deep vein thrombosis (excluding thrombophlebitis); arterial thrombosis; peripheral artery disease; thrombocytosis; post-heart transplant. |
| Major systemic comorbidity | Congestive heart failure, cerebrovascular, neoplastic, and chronic liver or renal diseases, other chronic cardiovascular and neurologic conditions (except hypertension), diabetes mellitus, autoimmune disorders, immunocompromised. |
| Other comorbidities | - Renal failure: chronic renal dysfunction and/or dialysis. - Endocrinal diseases: diabetes mellitus; exocrine pancreas insufficiency (associated with CF); hyper- or hypothyroidism; pan hypopituitarism; Addison’s disease. - Liver diseases: liver cirrhosis; active hepatitis; Gilbert’s disease. - Human immunodeficiency virus (HIV). - (Disabling) neurological diseases: myasthenia gravis; dementia; Parkinson’s disease; polyneuropathy; neurosarcoidosis. - Rheumatological: systemic lupus erythematosus (SLE); Sjögren’s disease; Bechterew’s disease. - Solid tumours (current & history): malignant solid tumours (excluding basal cell carcinoma). - Hematologic diseases (current & history): polycythaemia vera; haematological cancer; idiopathic thrombocytopenic purpura (ITP); hemochromatosis. |
| Smoking | Smoking currently and/or smoking in the past for at least 5 years. |
| Confusion | Having a Glasgow Coma Scale <15 and/or statement of lowered consciousness at physical examination. |
| Lower respiratory tract infection/ pneumonia | New pulmonary infiltrative abnormalities on a patient’s chest radiograph (X-ray or CT-scan). |
| Bacterial coinfection/ superinfection | Urine antigen test for *S. pneumoniae* and *L. pneumophilia* positive and/or sputum culture positive with >10 CFU for any bacterium (+/- blood culture positive with >10 CFU for bacterium) and/or blood sample. |
| Requirement mechanical ventilation | Persistent respiratory failure despite supplemental oxygen therapy necessitating the use of non-invasive positive pressure ventilation (NIPPV) or invasive mechanical ventilation for support. |

**Supplementary Figure 1.** *Absolute numbers of included patients and patients who died during their hospital stay, per calendar year during inclusion period (January 2005 through April 2018).*

* 2018 includes only 4 months (January through April).

**Supplementary Figure 2.** *Flow chart search MEDLINE and screening.*

^a^ Search terms: [respiratory syncytial virus OR RSV] AND [prognosis OR prognostic] AND [adult OR adults].

^b^ We excluded studies that developed prognostic models with disease progression to lower respiratory tract infections as primary outcome.
